# Loss of heterozygosity exploited for collateral lethality-based cancer therapy

**DOI:** 10.1016/j.ebiom.2024.105388

**Published:** 2024-10-08

**Authors:** Hai Fang

**Affiliations:** Shanghai Institute of Hematology, State Key Laboratory of Medical Genomics, National Research Center for Translational Medicine at Shanghai, Ruijin Hospital, Shanghai Jiao Tong University School of Medicine, Shanghai 200025, China

As a common genetic event during cancer development, loss of heterozygosity (LOH) results in the loss of one allele of a gene, leading to reduced genetic diversity. In certain tumor types, this loss can affect a substantial portion of the genome, creating distinct genetic differences between tumor cells and their normal counterparts. These genetic alterations can be therapeutically exploited to induce cancer-specific cell death, a concept known as ‘collateral lethality’.^1^ Therapies based on this concept have emerged as a promising strategy for developing more precise treatments by capitalising on the loss of specific gene functions in tumors. The first crucial step in this strategy is the identification of genetic alterations specific to tumor cells that can be selectively targeted. In this issue of *eBioMedicine*, Zhang and colleagues have made significant progress toward this goal by identifying key genetic targets and therapeutic strategies,^2^ contributing to the growing watchlist of therapeutic targets that may shape the future of collateral lethality-based therapy targeting LOH in cancer (Fig. 1).

**Fig. 1:**
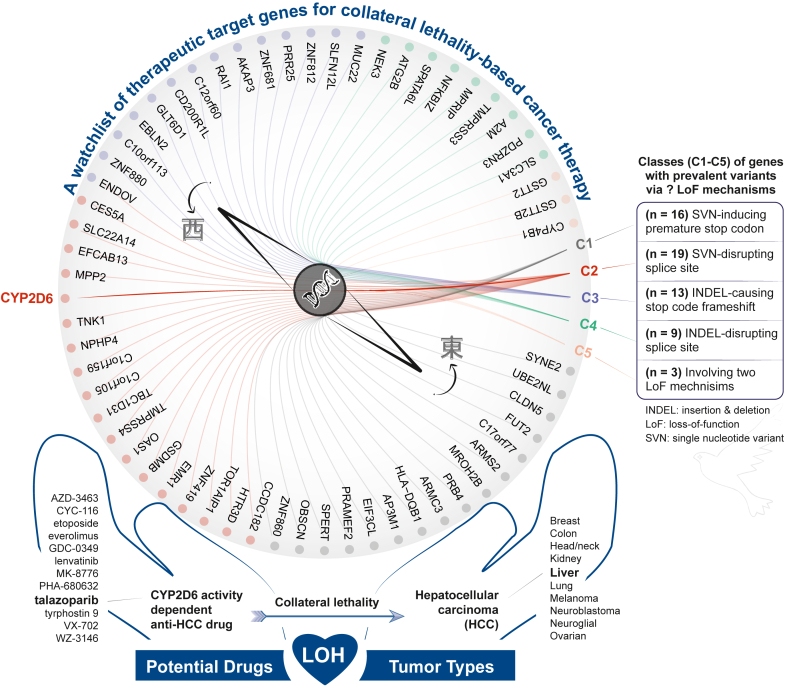
**Collateral lethality-based therapy targeting loss of heterozygosity (LOH) in hepatocellular carcinoma (HCC)**. The compass-like diagram in the upper panel circularly displays 60 therapeutic target genes with prevalent loss-of-function (LoF) variants, categorised by their LoF mechanisms into five classes (C1–C5; color-coded). Among these, the gene CYP2D6 is highlighted as an exemplary target by Zhang and colleagues. They discovered 12 potential drugs, with one drug talazoparib showing CYP2D6 genotype-dependent anti-HCC effects (the lower panel). Their findings show how exploiting the loss of drug-metabolising enzyme gene activity in tumor cells following LOH can offer a promising therapeutic strategy. In this *Commentary*, Fang comments on the significance of these findings from both basic and clinical research perspectives, discusses their potential to transform current clinical practices and inspire future research directions into LOH-directed cancer therapy, and concludes with a perspective on genetics-led approaches to precision medicine. Genetically inspired research should be explored in all directions, without being restricted by the east-or-west directions indicated by compass pointers.

Zhang et al. mined loss-of-function (LoF) alleles in cancer genomes, identifying 60 target genes grouped into five classes based on their predicted LoF mechanisms. Among these, CYP2D6 stands out as a particularly promising target due to its frequent LOH in multiple tumor types, including hepatocellular carcinoma (HCC). Using engineered cell models, they identified 12 potential drugs capable of exploiting this LOH. Notably, the clinically available drug called ‘talazoparib’ showed significant potential by exhibiting CYP2D6 genotype-dependent anti-HCC effects. This study demonstrates how the loss of drug-metabolising enzymes in tumors can be harnessed by existing approved drugs, enabling drug repurposing in cancers with unmet medical needs.

The implications are profound for both basic and clinical research. For basic scientists, identifying genes impacted by LOH opens new avenues for understanding cancer's genetic complexity. Targeting specific genes (e.g. CYP2D6) based on their LOH provides an opportunity to explore how drug-metabolising enzymes contribute to tumorigenesis. For clinical scientists, identifying drugs (e.g. talazoparib) that rely on these genes introduces significant possibilities for personalised treatment strategies, especially in cancers (e.g., HCC) where LOH is common. This study highlights the potential for genetic profiling to guide clinical decision-making. By identifying patients whose tumors exhibit LOH in genes like CYP2D6, clinicians can tailor treatment plans to exploit these genetic vulnerabilities. This approach also encourages future clinical trials to assess the efficacy of therapies that depend on gene activity, using both the patient's constitutional and tumor genotypes as a guide.

Incorporating genetic profiling into current clinical practices to detect LOH in specific genes, such as CYP2D6, could guide personalised medicine. For instance, gene panel sequencing (or whole-exome sequencing) could be used to diagnose patients with HCC (or with liver disease of unknown etiology^3^), helping clinicians identify patients who are most likely to benefit from targeted therapies. The potential to repurpose approved drugs (e.g. talazoparib) for new therapeutic use is particularly promising, as it offers a cost-effective way to improve outcomes for patients with limited treatment options. However, further clinical validation in larger patient cohorts is necessary to establish clear guidelines on the safety, efficacy, and optimal dosing regimens of these repurposed therapies, as well as their interactions with established treatments.

Despite the significant progress summarised above, several unanswered questions remain for future research. For basic scientists, the prevalence and relevance of CYP2D6 LOH across different cancer types are still unclear. It is yet to be determined whether this phenomenon is widespread or confined to specific cancers. Furthermore, the broader role of drug-metabolising enzymes in cancer progression requires further elucidation, particularly with regard to metabolism-altering cancer mutations.^4^ Understanding how LOH in other enzymes contributes to drug sensitivity or resistance could uncover new therapeutic opportunities. From a clinical standpoint, unanswered questions regarding the long-term safety and efficacy of talazoparib in patients with CYP2D6 LOH remain to be addressed. These include determining optimal dosages, understanding how these therapies interact with other treatments, and identifying specific subgroups of patients who are more likely to benefit from collateral lethality-based therapy targeting LOH.

In conclusion, Zhang et al. exemplify how genetic alterations in cancer can be leveraged to discover therapeutic targets and repurpose existing drugs. Although further validation in clinical settings is required, their findings pave the way for therapeutic innovation. Exploiting LOH for therapeutic purposes marks a significant advancement in cancer therapy, particularly given its prior applications in cancer chemotherapy^5^ and immunotherapy.^6^ Furthermore, its similar concept known as ‘synthetic lethality’ has long driven large-scale genetic screens for anti-cancer drug targets.^7^^,^^8^ More broadly, genetics-led approaches — identifying naturally occurring genetic alterations and then evaluating the effects of perturbations in genetic target candidates — align well with the philosophy of ‘clinical trials by nature’. Here, naturally occurring genetic variations inform target prioritisation and mechanism discovery,^9^ guiding the discovery of genetic targets for clincial success.^10^ By harnessing LOH and natural genetic alterations, new treatment avenues can be uncovered, fostering the development of targeted therapies tailored to patients' specific genetic profiles, thereby accelerating the advancement of precision medicine in cancer and beyond.

## Contributors

H.F. performed the literature search, designed the figure, and wrote this Commentary.

## Declaration of interests

The author declares no competing interests.
